# Indol as an Etiologic Factor in Arteriosclerosis

**Published:** 1912-12

**Authors:** 


Indol as an Etiologic Factor in Arteriosclerosis.—At the Metchnikoff Laboratory in Paris, Dratschinski (*Ann. de L' Institut Pasteur,* June, 1912), has injected indol into guinea pigs to determine its toxic action, and found that symptoms of severe auto-intoxication were produced after very moderate doses, attacking chiefly the peripheral nervous system and the muscles. If continued for any length of time, .04 gm. by mouth produces atheromatous changes in the aorta and brain, cirrhosis of the liver and adrenals, which may progress and have a fatal outcome. Similar changes were observed in monkeys, rats and mice. One may naturally conclude that indol is a factor in the production of the sclerotic changes in man, and it is to be hoped that further researches will be carried on along this line.

